# Dipeptidyl peptidase 4 inhibitor use is associated with a lower risk of incident acute kidney injury in patients with diabetes

**DOI:** 10.18632/oncotarget.18081

**Published:** 2017-05-23

**Authors:** Chia-Ter Chao, Jui Wang, Hon-Yen Wu, Kuo-Liong Chien, Kuan-Yu Hung

**Affiliations:** ^1^ Department of Medicine, National Taiwan University Hospital Jin-Shan Branch, New Taipei City, Taiwan; ^2^ Department of Internal Medicine, National Taiwan University Hospital and College of Medicine, Taipei, Taiwan; ^3^ Institute of Epidemiology and Preventive Medicine, College of Public Health, National Taiwan University, Taipei, Taiwan; ^4^ Department of Internal Medicine, Far-Eastern Memorial Hospital, New Taipei City, Taiwan; ^5^ Faculty of Medicine, School of Medicine, National Yang-Ming University, Taipei, Taiwan; ^6^ Department of Internal Medicine, National Taiwan University Hospital Hsin-Chu Branch, Hsin-Chu City, Taiwan

**Keywords:** acute kidney injury, dialysis-requiring acute kidney injury, chronic kidney disease, diabetes mellitus, dipeptidyl peptidase 4 inhibitor

## Abstract

**Objectives:**

Dipeptidyl peptidase 4 inhibitor (DPP4i) use potentially slows the progression of diabetic kidney disease, but its effects on the risk of acute kidney injury (AKI) are unclear. We aimed to assess the association between DPP4i use and incident AKI episodes from a nationally representative cohort in Taiwan.

**Materials and Methods:**

All patients newly diagnosed with diabetes mellitus (DM) between 2008, when DPP4i use was first approved in Taiwan, and mid-2013 were enrolled. Propensity score-matched diabetic DPP4i users, who received DPP4i for at least 90 days, and nonusers were selected. The primary and secondary outcomes were incident AKI and dialysis-requiring AKI during follow-up. Cox proportional hazard analyses were performed to examine the effect of DPP4i on the risk of AKI.

**Results:**

We enrolled 923,936 diabetic patients; of these, 83,638 DPP4i users (75.7% sitagliptin, 14.6% vildagliptin, and 9.7% saxagliptin) were propensity score-matched to 83,638 non-users. After an average 3.6-year follow-up, 1.56% and 0.35% of DPP4i users and 2.53% and 0.56% of non-users developed incident AKI and dialysis-requiring AKI, respectively. DPP4i use was significantly associated with lower risk of incident AKI (hazard ratio [HR] 0.57, 95% confidence interval [CI] 0.53–0.61) and risk of dialysis-requiring AKI (HR 0.57, 95% CI 0.49–0.66). The risk reduction was consistent regardless of DPP4i type, the presence of chronic kidney disease, the previous acute kidney injury, and age.

**Conclusions:**

DPP4i use is associated with reduced risk of mild and severe forms of AKI among patients with incident DM. DPP4i may be an important class of anti-glycaemic agent with reno-protective effects.

## INTRODUCTION

Diabetes mellitus (DM) is an important public health concern that results in an increased risk of adverse health events, among which diabetic kidney disease (DKD) is highly prevalent [1]. DKD is responsible for nearly half of the end-stage renal disease (ESRD) cases in developing and developed countries [2]. The pathogenesis of DKD involves glucotoxicity from advanced glycation end-products, local renin-angiotensin system activation, and altered redox balance, but the renal influences of anti-glycaemic agents remain controversial [3].

Dipeptidyl peptidase 4 inhibitor (DPP4i), a new incretin-based anti-diabetic medication, lowers glycated haemoglobin (HbA1c) levels by an average of 0.62% to 0.85% and carries a lower risk of hypoglycaemia compared to other anti-diabetic medications [4]. This glucose-lowering effect is achieved through suppression of the inactivation of glucagon-like-peptide 1 (GLP-1), leading to reduced glucagon release, delayed gastric emptying, and increased β-cell survival. Growing evidence suggests that GLP-1 agonists could retard the progression of DKD by ameliorating inflammation and fibrosis and improving endothelial functions [5, 6]. DPP4 is widely distributed in endothelial and epithelial tissues, including renal proximal tubular epithelia, podocytes, mesangial cells, and pre-glomerular vascular smooth muscle cells [7]. In addition, diabetic animals reportedly have increased renal DPP4 expression, and DPP4 knockout status predisposes diabetic rats to subsequent renal function impairment [8]. Based on these findings, DPP4 might play an important role in the development of DKD, and DPP4i may potentially slow the progression of CKD in diabetic patients.

Several experimental and small cohort studies have revealed that vildagliptin and linagliptin exert reno-protective effects via the reduction of albuminuria [9, 10]. As the presence of CKD is an important risk and predisposing factor for the development of acute kidney injury (AKI), it is likely that DPP4i plays an under-recognized role in modifying the risk of developing AKI. A group of researchers recently evaluated whether DPP4i use was associated with altered risk of AKI, using a case-control design [11]. They found that among 6,752 patients with AKI and their matched control, the former group were 20% more likely to be DPP4i users with the past year of their AKI episode than the latter. However, this report is limited to due to its study design, which does not permit any causal inference. To clarify the exact relationship between DPP4i use and the risk of developing AKI, we used data from the Taiwan National Health Insurance (NHI) database and designed a prospective cohort study to help clarify whether DPP4i use can influence the risk of subsequent AKI.

## RESULTS

**Figure 1:**
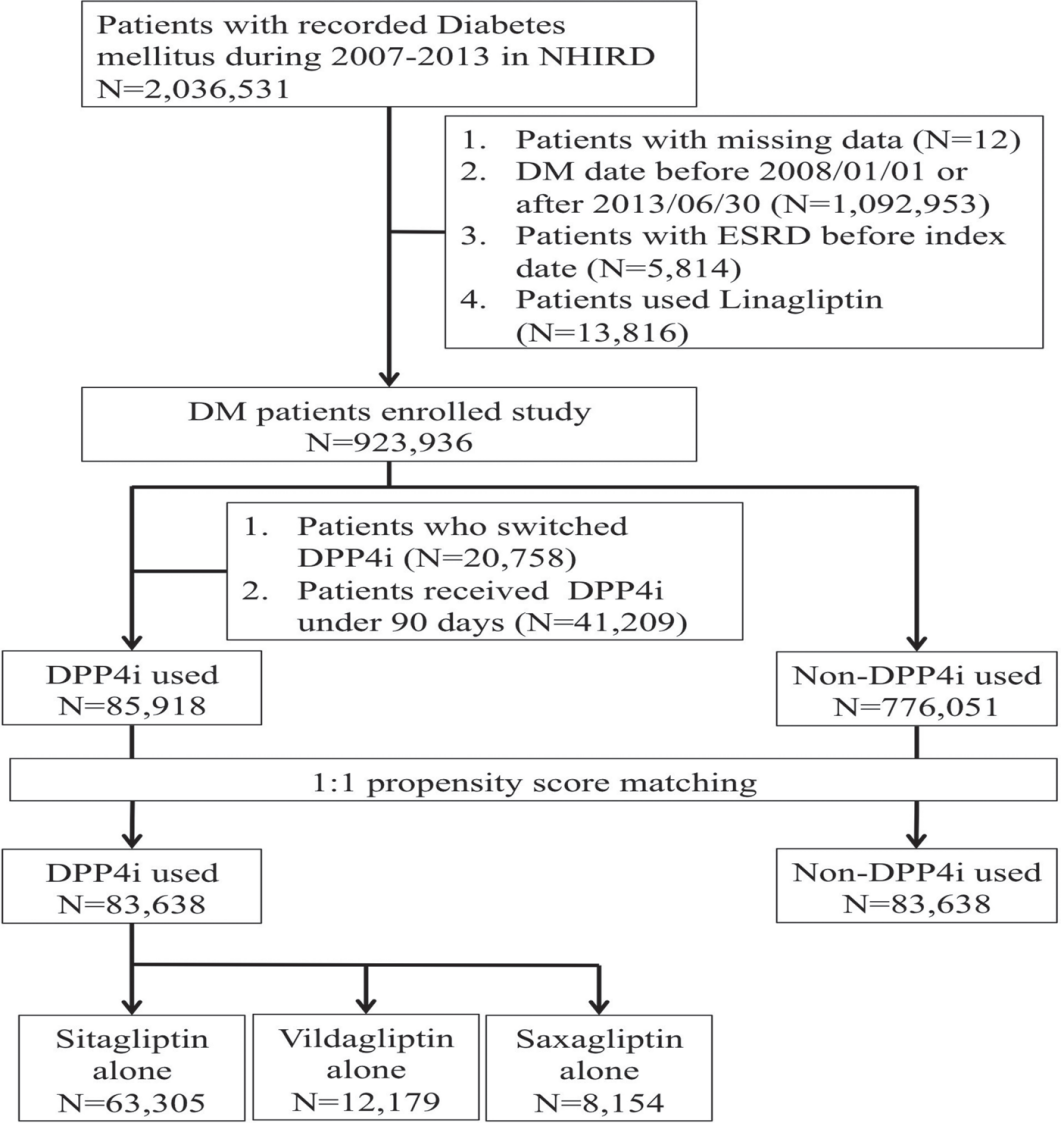
Flow diagram of the current study ESRD, end-stage renal disease; DPP4i, dipeptidyl peptidase 4 inhibitor; NHIRD, National Health Insurance Research Database.

A total of 2,036,531 participants with a diagnosis of DM were identified and recruited (Figure 1). A period of six months was allowed for the development of the outcome of interest after diagnosis, so those participants diagnosed with DM after June 30, 2013, were excluded. Finally, 923,936 DM patients were enrolled, among whom 83,638 DPP4i users and propensity score-matched non-users in a 1:1 ratio were included in the analysis, excluding those who switched between DPP4i classes and who did not receive DPP4i for three months consecutively. Among users, 75.7% used sitagliptin, followed by vildagliptin (14.6%) and saxagliptin (9.7%) (Figure 1).

There was no significant difference between DPP4i users and non-users regarding the 40 measured baseline characteristics, including age, sex, comorbidities, Charlson comorbidity index, healthcare uses with potential renal influences before enrolment, and concurrent medications (Table 1). A higher proportion of DPP4i users were urban residents and were more likely to receive clopidogrel and ezetimibe during the study period compared to non-users. For anti-diabetic medications, DPP4i users were more likely to receive insulin, while non-users more frequently received sulfonylurea and biguanide (Table 1).

**Table 1:** Clinical features of diabetic participants with and without DPP4 inhibitor treatment

Variables	DPP4i users (*n* = 83,638)	DPP4i non-users (*n* = 83,638)	*p* value
*Demographic and socioeconomic profiles*			
Age (years)	55.8 ± 12.8	55.9 ± 12.8	0.99
Gender (female %)	36457 (43.6)	36221 (43.3)	0.24
Residential location (urban %)	48689 (58.2)	48224 (57.7)	0.02
*Comorbidities*			
Hypertension (%)	43456 (52)	43616 (52.1)	0.43
Hyperlipidemia (%)	31483 (37.6)	31311 (37.4)	0.39
Chronic liver disease (%)	12304 (14.7)	12310 (14.7)	0.97
Atrial fibrillation (%)	4665 (5.6)	4658 (5.6)	0.94
Chronic obstructive pulmonary disease (%)	3152 (3.8)	3208 (3.8)	0.47
Acute coronary syndrome (%)	10108 (12.1)	9931 (11.9)	0.18
Cerebrovascular disease (%)	7587 (9.1)	7648 (9.1)	0.60
Malignancy (%)	3437 (4.1)	3439 (4.1)	0.98
Parkinsonism (%)	622 (0.7)	648 (0.8)	0.46
Chronic kidney disease (%)	7732 (9.2)	7880 (9.4)	0.21
Advanced chronic kidney disease (%)	38 (0.05)	31 (0.04)	0.40
Past experience of AKI (%)	828 (1)	881 (1.1)	0.20
Peripheral vascular disease (%)	793 (0.9)	788 (0.9)	0.90
Benign prostatic hyperplasia (%)	3936 (4.7)	3956 (4.7)	0.82
*Charlson comorbidity index*	*1.64 ± 1.3*	*1.64 ± 1.3*	*0.69*
*Intervention before the date of enrollment*			
Computed tomography of any site (%)	6138 (7.3)	6123 (7.3)	0.89
Cardiac catheterization (%)	1555 (1.9)	1559 (1.9)	0.94
Angiography of any site (%)	1306 (1.6)	1291 (1.5)	0.77
Cystoscopy with or without biopsy (%)	1317 (1.6)	1356 (1.6)	0.45
Transurethral resection of prostate (%)	171 (0.2)	174 (0.2)	0.87
*Chronic medication use*			
Aspirin (%)	31265 (37.4)	30923 (37.0)	0.08
β-blocker (%)	36654 (43.8)	36473 (43.6)	0.37
ACEI (%)	19820 (23.7)	20060 (24.0)	0.17
ARB (%)	47154 (56.4)	47030 (56.2)	0.54
Clopidogrel (%)	6881 (8.2)	6558 (7.8)	<0.01
Statin (%)	54533 (65.2)	54355 (65.0)	0.36
NSAID (%)	76722 (91.7)	76764 (91.8)	0.71
COX2 inhibitor (%)	20379 (24.4)	20188 (24.1)	0.28
Fibrate (%)	20441 (24.4)	20523 (24.5)	0.64
Ezetimibe (%)	8224 (9.8)	7526 (9.0)	< 0.01
Calcium channel blocker (%)	46073 (55.1)	46132 (55.2)	0.77
α-blocker (%)	9082 (10.9)	9035 (10.8)	0.71
Wafarin (%)	1979 (2.4)	1944 (2.3)	0.57
Platinum-based anti-neoplastic agents (%)	1036 (1.2)	1078 (1.3)	0.36
Atypical anti-psychotics (%)	2887 (3.5)	2877 (3.4)	0.89
Nephrotoxic anti-bacterial agents (%)*	6027 (7.2)	5969 (7.1)	0.58
Nephrotoxic anti-viral agents (%)^&^	915 (1.1)	959 (1.1)	0.31
Cyclosporin and tacrolimus (%)	94 (0.1)	91 (0.1)	0.83
Lithium (%)	204 (0.2)	218 (0.3)	0.50
*Anti-diabetic medications*			
Insulin (%)	15293 (18.3)	14881 (17.8)	0.01
Sulfonylurea (%)	64866 (77.6)	65529 (78.3)	< 0.01
Biguanides (%)	78354 (93.7)	79330 (94.8)	< 0.01

Data are expressed as mean ± standard deviation for continuous variable and number (percentage) for categorical variables

*Including vancomycin, aminoglycosides, and colistin

^&^Including acyclovir, and ganciclovir

ACEI, angiotensin-converting enzyme inhibitor; AKI, acute kidney injury; ARB, angiotensin receptor blocker; COX2, cyclo-oxygenase 2; DPP4i, dipeptidyl peptidase 4 inhibitor; NSAID, non-steroidal anti-inflammatory drug.

**Figure 2:**
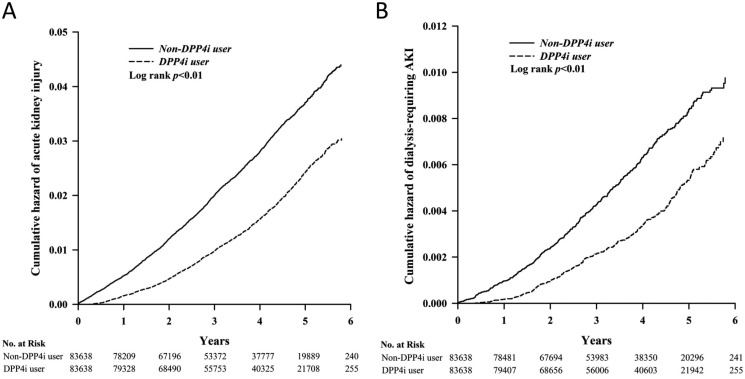
Kaplan-Meier cumulative hazard curve for incident AKI (**A**) and incident dialysis-requiring AKI (**B**) among study participants. AKI, acute kidney injury; DPP4i, dipeptidyl peptidase 4 inhibitor.

**Figure 3:**
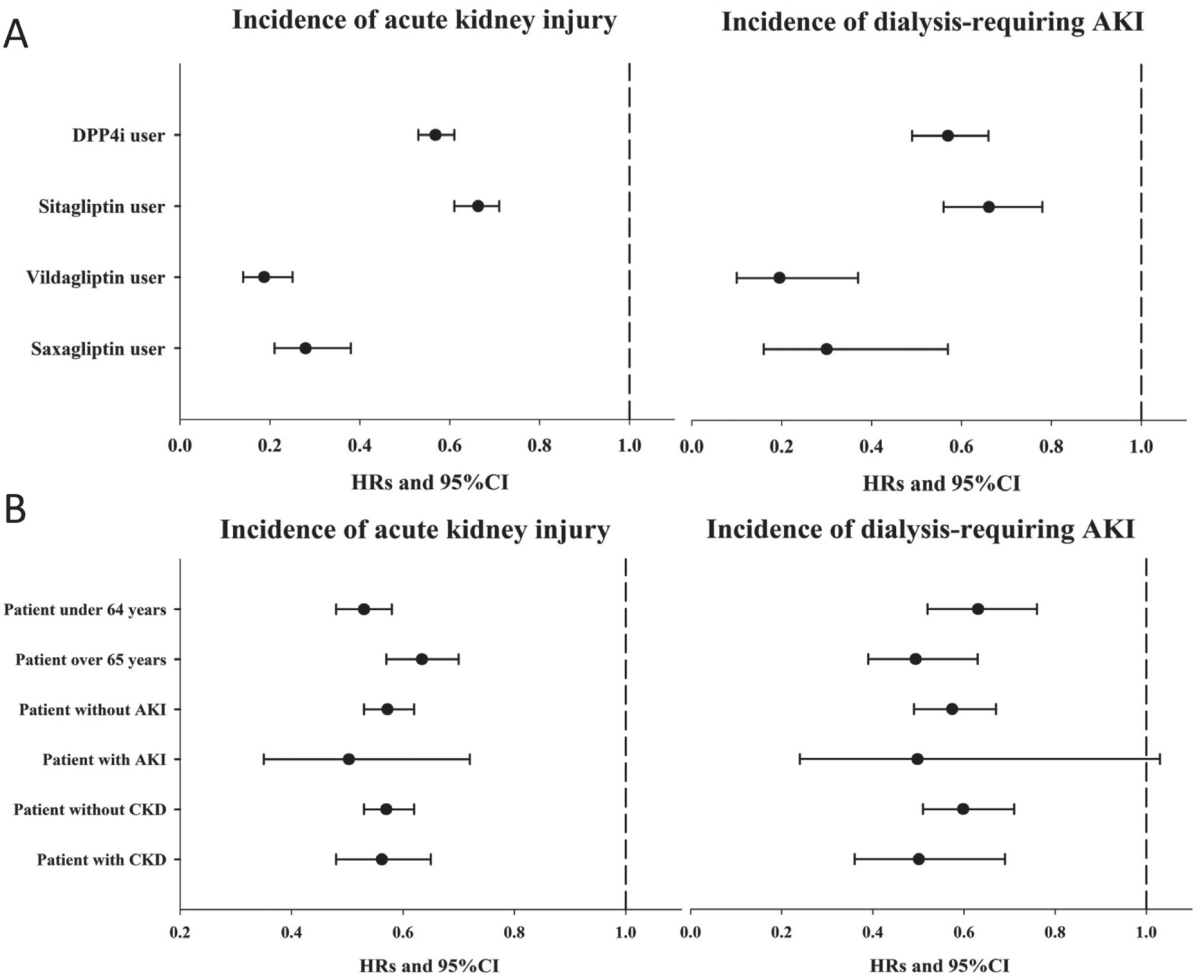
Forest plots illustrating the hazard ratio for incident AKI and for incident dialysis-requiring AKI among different DPP4i members (**A**) and pre-specified age and co-existing illnesses subgroups (**B**). AKI, acute kidney injury; CI, confidence interval; CKD, chronic kidney disease; DPP4i, dipeptidyl peptidase 4 inhibitor; HR, hazard ratio

For the primary (incident AKI) and secondary (incident dialysis-requiring AKI) outcomes, we observed DPP4i use was associated with a significantly lower risk of AKI after an average of 3.6 years of follow-up (2,118 episodes during 299,800 person-years [7.1 episodes/1,000 person-years] vs. 1,304 episodes during 309,331·9 person-years [4.2 episodes/1,000 person-years], HR 0.59 [95% CI, 0.55 to 0.63]) (Table 2) compared to non-users, as demonstrated by Kaplan-Meier cumulative hazard curves (Figure 2). After accounting for age, sex, comorbidities, interventions, and medications used during the study period, the risk reduction remained significant (HR 0.57; 95% CI, 0.53 to 0.61). Analyses of secondary outcomes also showed DPP4i use to be associated with significantly lower risk of dialysis-requiring AKI compared to non-users (469 episodes during 302,235 person-years [1.6 episodes/1,000 person-years] vs. 289 episodes during 310,358.4 person-years [0.9 episodes/1,000 person-years], HR 0.60; 95% CI, 0.51 to 0.69); the risk reduction also remained significant after multivariate adjustment (Table 2). All DPP4i members of interest exhibited a similar trend for lower risk of AKI and dialysis-requiring AKI versus non-users, with lower HRs for vildagliptin and saxagliptin users (Table 2 and Figure 3A). The cumulative hazard curves for each DPP4i examined in this study are shown in Supplementary Figure 1.

**Table 2:** Incidence and risk of acute kidney injury associated with different DPP4 inhibitor use among diabetic participants

				Crude Model†		Fully adjusted Model*	
Variables	Number of event	Duration (person-years)	Incidence density (per 1000 year)	HR	95% CI	HR	95% CI
*Incidence of acute kidney injury*							
Non-DPP4i users	2118	299800.0	7.1	1.00	-	1.00	-
DPP4i users	1304	309331.9	4.2	0.59	0.55 ∼ 0.63^a^	0.57	0.53 ∼ 0.61^a^
Non-DPP4i users	1623	227513.2	7.1	1.00	-	1.00	-
Sitagliptin users	1191	238378.6	5.0	0.69	0.64 ∼ 0.75^a^	0.66	0.61 ∼ 0.71^a^
Non-DPP4i users	291	43454.3	6.7	1.00	-	1.00	-
Vildagliptin users	54	42360.3	1.3	0.19	0.14 ∼ 0.25^a^	0.19	0.14 ∼ 0.25^a^
Non-DPP4i users	204	28832.6	7.1	1.00	-	1.00	-
Saxagliptin users	59	28593.0	2.1	0.29	0.22 ∼ 0.39^a^	0.28	0.21 ∼ 0.38^a^
*Incident dialysis-requiring acute kidney injury*							
Non-DPP4i users	469	302235.0	1.6	1.00	-	1.00	-
DPP4i users	289	310358.4	0.9	0.60	0.51 ∼ 0.69^a^	0.57	0.49 ∼ 0.66^a^
Non-DPP4i users	363	229363.8	1.6	1.00	-	1.00	-
Sitagliptin users	265	239362.7	1.1	0.69	0.59 ∼ 0.81^a^	0.66	0.56 ∼ 0.78^a^
Non-DPP4i users	63	43807.5	1.4	1.00	-	1.00	-
Vildagliptin users	12	42376.9	0.3	0.20	0.11 ∼ 0.37^a^	0.20	0.10 ∼ 0.37^a^
Non-DPP4i users	43	29063.7	1.5	1.00	-	1.00	-
Saxagliptin users	12	28618.8	0.4	0.28	0.15 ∼ 0.53^b^	0.30	0.16 ∼ 0.57^b^

^a^ *p* < 0.0001; ^b^ *p* < 0.01

† Calculated by stratified Cox proportional regression

* Adjusted for age, gender, all comorbidities, and medications

CI, confidence interval; DPP4i, dipeptidyl peptidase 4 inhibitor; HR, hazard ratio.

The results of subgroup analyses are shown in Supplementary Tables 1, 2, and 3. The presence of CKD (Supplementary Table 1), history of AKI (Supplementary Table 2), and age (Supplementary Table 3) did not influence the observed association (Figure 3B). However, compared to non-users, DPP4i use in diabetic patients without past AKI was not associated with lower incidence of dialysis-requiring AKI, irrespective of DPP4i type (Supplementary Table 2). Owing to the short follow-up periods and low event numbers in the saxagliptin group, the association was insignificant among saxagliptin users without CKD but remained significant among sitagliptin and vildagliptin users without CKD (Supplementary Table 1). Finally, we also evaluated the influence of DPP4i dosage on the primary and secondary outcomes (Table 3). Users receiving higher sitagliptin doses had progressively lower risks of incident AKI and dialysis-requiring AKI compared to those receiving lower doses, while higher vildagliptin and saxagliptin doses were associated with lower risks of incident AKI and dialysis-requiring AKI, respectively. Since sitagliptin, vildagliptin, and saxagliptin doses need to be reduced in patients with CKD, we further excluded those with CKD and repeated the analyses. We found that among those without CKD, users of higher sitagliptin dose still had a significantly lower risk of developing incident AKI (HR 0.72 and 0.37 for those within the middle and the highest tertile, 95% CI 0.62–0.84 and 0.31–0.44, respectively), compared to those within the lowest tertile (Table 3). Similar findings were also observed for vildagliptin users without CKD, but not for saxagliptin users without CKD (Table 3).

**Table 3:** Incidence and risk of acute kidney injury associated with DPP4 inhibitor use stratified by defined daily dose (DDD)

					Crude Model†		Fully adjusted Model*
Variables	Number of event	Duration (person-years)	Incidence density (per 1000 year)	HR	95% CI	HR	95% CI
*Incidence of acute kidney injury*							
**Sitagliptin users**							
lowest tertile	497	76453.6	6.5	1.00	-	1.00	-
middle tertile	378	77086.5	4.9	0.75	0.66 ∼ 0.86^a^	0.81	0.71 ∼ 0.93^b^
highest tertile	316	84850.4	3.7	0.56	0.48 ∼ 0.64^a^	0.65	0.56 ∼ 0.75^a^
**Vildagliptin users**							
Under median	41	21159.1	1.9	1.00	-	1.00	-
Over median	13	21201.2	0.6	0.31	0.17 ∼ 0.58^b^	0.33	0.17 ∼ 0.63^b^
**Saxagliptin users**							
Under median	39	14637.3	2.7	1.00	-	1.00	-
Over median	20	13955.6	1.4	0.54	0.31 ∼ 0.92^c^	0.66	0.38 ∼ 1.14
*Incidence of acute kidney injury: excluding those with CKD*							
**Non-CKD Sitagliptin users**							
lowest tertile	394	61919.7	6.4	1.00	-	1.00	-
middle tertile	267	60525.1	4.4	0.68	0.58 ∼ 0.79^a^	0.72	0.62 ∼ 0.84^a^
highest tertile	170	75999.9	2.2	0.32	0.27 ∼ 0.39^a^	0.37	0.31 ∼ 0.44^a^
**Non-CKD Vildagliptin users**							
Under median	26	17515	1.5	1.00	-	1.00	-
Over median	8	17986.9	0.4	0.29	0.13 ∼ 0.65^b^	0.32	0.14 ∼ 0.72^b^
**Non-CKD Saxagliptin users**							
Under median	17	11963.2	1.4	1.00	-	1.00	-
Over median	15	11772.2	1.3	0.88	0.44 ∼ 1.76	0.91	0.45 ∼ 1.85
*Incident dialysis-requiring acute kidney injury*							
**Sitagliptin users**							
lowest tertile	109	76913.5	1.4	1.00	-	1.00	-
middle tertile	87	77370.7	1.1	0.79	0.60 ∼ 1.05	0.84	0.63 ∼ 1.12
highest tertile	69	85090.4	0.8	0.56	0.41 ∼ 0.75^b^	0.64	0.47 ∼ 0.87^b^
**Vildagliptin users**							
Under median	9	21172.7	0.4	1.00	-	1.00	-
Over median	3	21204.1	0.1	0.33	0.09 ∼ 1.22	0.26	0.06 ∼ 1.21
**Saxagliptin users**							
Under median	11	14652.0	0.8	1.00	-	1.00	-
Over median	1	13966.9	0.1	0.10	0.01 ∼ 0.73^c^	0.09	0.01 ∼ 0.75^c^
*Incident dialysis-requiring acute kidney injury: excluding those with CKD*							
**Non-CKD Sitagliptin users**							
lowest tertile	85	62242.1	1.4	1.00	-	1.00	-
middle tertile	61	60733.8	1.0	0.72	0.52 ∼ 0.99^c^	0.74	0.53 ∼ 1.03
highest tertile	44	76115.8	0.6	0.39	0.27 ∼ 0.56^a^	0.43	0.3 ∼ 0.62^a^
**Non-CKD Vildagliptin users**							
Under median	5	17522.4	0.3	1.00	-	1.00	-
Over median	1	17989.4	0.1	0.19	0.02 ∼ 1.62	0.17	0.003 ∼ 9.78
**Non-CKD Saxagliptin users**							
Under median	7	11969.9	0.6	1.00	-	1.00	-
Over median	1	11780.1	0.1	0.14	0.02 ∼ 1.14	0.12	0.01 ∼ 1.04

^a^*p* < 0.0001; ^b^*p* < 0.01; ^c^*p* < 0.05

†Calculated by stratified Cox proportional regression

*Adjusted for age, gender, all comorbidities, and medications

CI, confidence interval; DPP4, dipeptidyl peptidase 4; HR, hazard ratio

Several post-hoc sensitivity analyses were done to validate our findings. First, we focused on those without past experiences of AKI to exclude the influence of prevalent cases. We found that DPP4i users without prior AKI still had significantly lower risk of developing incident AKI (HR 0.57, 95% CI 0.5 to 0.67) and incident dialysis-requiring AKI (HR 0.58, 95 CI 0.5 to 0.67) than non-users (Table 4). We also analysed only those with a MPR ≥ 80% during the first year after study entry, to restrict our analysis to continuous DPP4i users only. We found that among those with a MPR ≥ 80%, DPP4i users still had a significantly lower risk of developing incident AKI (HR 0.45, 95% CI 0.39–0.51) and incident dialysis-requiring AKI (HR 0.45, 95% CI 0.34 to 0.59), compared to non-users (Table 4). Accounting for diabetic severity by incorporating aDCIS scores in the Cox regression model, DPP4i users also had a significantly lower risk of developing incident AKI (HR 0.55, 95% CI 0.51–0.59) and incident dialysis-requiring AKI (HR 0.55, 95% CI 0.51–0.59) compared to non-users. Incorporating aDCIS complication counts in the Cox regression model yielded similar findings (for incident AKI, HR 0.57, 95% CI 0.49–0.66; for incident dialysis-requiring AKI, HR 0.56, 95% CI 0.48–0.65) compared to non-users (Table 4). If we focused only on those with a primary diagnosis of AKI as the outcome of interest, DPP4i users also had a significantly lower risk of developing incident AKI (HR 0.58, 95% CI 0.5 to 0.67) and incident dialysis-requiring AKI (HR 0.58, 95% CI 0.45–0.76), compared to non-users (Table 4). If we incorporated mortality as a competing risk in the Cox regression analyses, DPP4i users had a significantly lower risk of developing incident AKI (HR 0.6, 95% CI 0.56–0.64) and incident dialysis-requiring AKI (HR 0.6, 95% CI 0.52–0.69), compared to non-users (Table 4). Finally, if we included only those without CKD codes in our analyses, DPP4i users also had a significantly lower risk of developing incident AKI (HR 0.56, 95% CI 0.52–0.61) and incident dialysis-requiring AKI (HR 0.61, 95% CI 0.51–0.73), compared to non-users.

**Table 4:** Results from the sensitivity analyses

					Crude Model†		Fully adjusted Model*
**Variables**	**Number of event**	**Duration (person-years)**	**Incidence density (per 1000 year)**	**HR**	**95% CI**	**HR**	**95% CI**
*Incidence of acute kidney injury*							
**Excluding patients with past experiences of AKI**							
Non-DPP4i users	2001	294428.9	6.8	1.00	-	1.00	-
DPP4i users	1227	303872.1	4.0	0.59	0.55 ∼ 0.63^a^	0.57	0.53 ∼ 0.61^a^
**Medication Possession Ratio ≥ 80%: first year**							
Non-DPP4i users	686	91932.6	7.5	1.00	-	1.00	-
DPP4i users	344	100161.5	3.4	0.45	0.4 ∼ 0.51^a^	0.45	0.39 ∼ 0.51^a^
**Incorporating aDCIS scores in analyses**							
Non-DPP4i users	2118	299800.0	7.1	1.00	-	1.00	-
DPP4i users	1304	309331.9	4.2	0.59	0.55 ∼ 0.63^a^	0.55	0.51 ∼ 0.59^a^
**Incorporating aDCIS complication counts in analyses**							
Non-DPP4i users	2118	299800.0	7.1	1.00	-	1.00	-
DPP4i users	1304	309331.9	4.2	0.59	0.55 ∼ 0.63^a^	0.57	0.49 ∼ 0.66^a^
**Including only those with AKI as the primary diagnosis**							
Non-DPP4i users	474	301965.6	1.6	1.00	-	1.00	-
DPP4i users	299	310259.2	1.0	0.61	0.53 ∼ 0.7^a^	0.58	0.5 ∼ 0.67^a^
**Death as a competing risk**							
Non-DPP4i users	2118	299800	7.1	1.00	-	1.00	-
DPP4i users	1304	309331.9	4.2	0.6	0.56 ∼ 0.65^a^	0.6	0.56 ∼ 0.64^a^
*Incident dialysis-requiring acute kidney injury*							
**Excluding patients with past experiences of AKI**							
Non-DPP4i users	437	296724.4	1.5	1.00	-	1.00	-
DPP4i users	273	304831.9	0.9	0.6	0.52 ∼ 0.7^a^	0.58	0.5 ∼ 0.67^a^
**Medication Possession Ratio ≥ 80%: first year**							
Non-DPP4i users	162	92677.7	1.7	1.00	-	1.00	-
DPP4i users	78	100407.8	0.8	0.44	0.33 ∼ 0.57^a^	0.45	0.34 ∼ 0.59^a^
**Incorporating aDCIS scores in analyses**							
Non-DPP4i users	469	302235.0	1.6	1.00	-	1.00	-
DPP4i users	289	310358.4	0.9	0.60	0.51 ∼ 0.69^a^	0.55	0.51 ∼ 0.59^a^
**Incorporating aDCIS complication counts in analyses**							
Non-DPP4i users	469	302235.0	1.6	1.00	-	1.00	-
DPP4i users	289	310358.4	0.9	0.60	0.51 ∼ 0.69^a^	0.56	0.48 ∼ 0.65^a^
**Including only those with AKI as the primary diagnosis**							
Non-DPP4i users	144	302529.4	0.5	1.00	-	1.00	-
DPP4i users	93	310492.9	0.3	0.62	0.48 ∼ 0.81^b^	0.58	0.45 ∼ 0.76^a^
**Death as a competing risk**							
Non-DPP4i users	469	302235	1.6	1.00	-	1.00	-
DPP4i users	289	310358.4	0.9	0.61	0.53 ∼ 0.7^a^	0.6	0.52 ∼ 0.69^a^

^a^ *p* < 0.0001; ^b^ *p* < 0.01

† Calculated by stratified Cox proportional regression

* Adjusted for age, gender, all comorbidities, and medications

CI, confidence interval; CKD, chronic kidney disease; DPP4, dipeptidyl peptidase 4; EPO, erythropoietin; HR, hazard ratio

## DISCUSSION

In this study, we demonstrated that the use of DPP4i in incident diabetic patients was associated with a significantly lower risk of incident AKI after treatment initiation compared with propensity-score matched diabetic controls. This effect was observed regardless of the severity of AKI as the study outcome, was consistent across different currently available types of DPP4i, and was more prominent with higher dosages. These novel findings may pave the way toward a better understanding of the renal effects of DPP4i for patients with DM.

The renal effect of conventional anti-diabetic medications has been evaluated intensively. A meta-analysis suggested that treatment with thiazolidinediones significantly attenuates the severity of albuminuria among users, while metformin and sulfonylurea have no such effect [12]. However, few studies have evaluated the renal effect of newer anti-diabetic medications, including DPP4i, and the existing reports focus mostly on albuminuria. Sitagliptin, alogliptin, and linagliptin use has been linked to a 16% to 30% lower urine albumin-to-creatinine ratio over four to 24 weeks, depending on the concurrent use of ARBs [10, 13]. Although DPP4i may ameliorate albuminuria in diabetic patients, clinical trials listing renal progression as the safety endpoint found minimal evidence for the renal benefits of DPP4i use. Furthermore, existing DPP4i-related studies rarely evaluate AKI as a primary endpoint. The findings from our nationwide cohort study contribute significantly to filling this knowledge gap by demonstrating the beneficial effect of DPP4i on the risk of AKI among patients with DM.

The relationship between DPP4i and the risk of AKI is rarely addressed. The Trial Evaluating Cardiovascular Outcomes with Sitagliptin (TECOS) study showed that the incidence of acute renal function decline did not differ between the DPP4i and control groups [14]. A pilot study retrospectively gleaned records of adverse renal events among clinical trials involving DPP4i, one of which was administrative code-detected AKI episodes [15]. The authors found that use of linagliptin conferred a favourable renal profile among 5,466 diabetic patients, while the risk of AKI remained neutral (HR 0.94, 95%CI 0.61 to 1.45) within months of follow-up. Others have also aimed to examine the renal effects of DPP4i, although their results are unavailable [16, 17]. Recently, another group from Taiwan reported that DPP4i use might be associated with higher risk of AKI among patients with type 2 diabetes mellitus, an opposite result [11]. However, they used a case-control design to examine the frequencies of DPP4i use among patients with and without AKI, potentially mistaking the effect for the cause. Moreover, they did not have follow-up results, and the case number was substantially smaller than ours. These methodologic inadequacies are important drawbacks for their findings, rendering interpretation of their results difficult. In contrast, our study has unique advantages compared to the existing literature. We incorporated three types of DPP4is in our analyses, used real-world data instead of highly selected trial participants, adjusted for comorbid illnesses, and enrolled substantially more patients (84,481) (Table 1). The benefits of DPP4i observed in the present study might be explained by the larger number of cases analysed in this population-based cohort, which allowed for greater statistical power; the longer duration of the follow-up period in each group (> 3 years vs. < 6 months in industry reports), and the ethnic composition of study participants. Interestingly, one study suggested that incretin-based therapies might be more efficacious in Asian diabetic patients than in Caucasian patients [18]. The possibility exists that the effect of DPP4i on AKI risk differs between patients of different ethnic origins, although further study is necessary for confirmation.

The renal effects of DPP4i might be explained directly by its influence on GLP-1 and the legacy of better glycaemic control, or indirectly by glucose-independent mechanisms such as an alteration in blood pressure control and weight change [7]. GLP1 receptor activation protects against renal ischemic-reperfusion injury (IRI) through haeme-oxygenase 1 induction, an important model of experimental AKI [19]. *In vitro* and preclinical data also suggest that DPP4i reduces the activity of sodium-hydrogen exchanger 3 (NHE3) in proximal tubules, resulting in increased urinary sodium excretion and potentially lower blood pressure [20]. However, DPP4 is an adipokine that is positively correlated with adiposity; pharmacological suppression of DPP4 increased peroxisome proliferator-activated receptor γcoactivator 1 (PPARGC1) expression, thereby reducing body fat content [21]. Dysglycaemia and hypertension have long been known to increase the risk of AKI in various clinical settings, while obesity and increased adiposity also predict higher incidence of AKI [22, 23]. Consequently, DPP4i might lower the risk of AKI through both glucose/GLP-1-related and glucose/GLP-1-unrelated pathways. Furthermore, a meta-analysis incorporating 18 randomised trials and nearly 5,000 participants concluded that DPP4i use is associated with 60% lower risk of acute coronary syndrome in diabetic patients [24]. This reduction in coronary events can also translate into a lower incidence of AKI due to cardiorenal syndrome. Indeed, experimental evidence already suggests that DPP4i pre-treatment can ameliorate renal ischemic-reperfusion injury in diabetic rats through its anti-inflammatory, anti-apoptotic, and anti-oxidative properties [25]. Our findings exemplify the protective effect of DPP4i against AKI in a real-world setting. Despite this benefit, studies showed that DPP4 deficient rats treated with streptozotocin have increased tendency toward developing dyslipidaemia and estimated glomerular filtration rate (eGFR) decline compared to their wild-type littermates; thus, the protective effect against AKI could be partially offset by the newly emerged lipid dys-regulation [8], a point that should be considered.

We found that individual members of DPP4i seemed to exhibit different degree of renoprotective effect (Figure 3). Sitagliptin had a numerically higher odds ratio (0.64) than the other DPP4i we studied (vildagliptin [0.22] and saxagliptin [0.39]), suggesting that sitagliptin might exert a relatively milder renoprotective effects than the other two. Past studies have identified that there could be intra-class differences for DPP4i members with regard to their biological influences. A meta-analysis on the potential cardiovascular benefits of DPP4i discovered that sitagliptin use was associated with a significantly lower risk of adverse cardiovascular events, while vildagliptin, saxagliptin, and alogliptin were not [24]. In SAVOR-TIMI 53 trial, saxagliptin use was found to increase the risk of heart failure hospitalization, while sitagliptin did not in TECOS trial [26, 27]. These findings serve as a plausible explanation for our results. In addition, the number of events and cohort size in the saxagliptin and vildagliptin groups was comparatively lower than those in the sitagliptin group; this issue could also affect the statistical power to detect the intensity of the relationship between DPP4i and the risk of AKI.

Dose-dependent analysis revealed that all three DPP4is evaluated in this study exhibited consistent protection against the development of AKI during follow-up (Figure 3). However, in subgroup analysis based on CKD or history of AKI, some of the examined DPP4is exhibited insignificant results in terms of benefit. Although lower event counts and case numbers are one explanation for this insignificant finding, individual differences exist may between members of the DPP4i class, although current evidence does not support this theory [28]. Trials of sitagliptin reported mildly increased incidence of adverse events including urinary tract infection and musculoskeletal complaint (arthralgia, back pain) among the treatment group compared to the control group [29], findings that have not been reported for newer member of the DPP4i family. Indeed, the affinity for DPP4 can vary significantly between different DPP4i. Nonetheless, our findings cannot be considered definitive, as the event numbers and case numbers are relatively low, especially among patient groups using the newer DPP4is. This issue may mask the true effects of the different DPP4is evaluated in this study.

### Limitations

Our study has its strengths, but there are also many limitations. The comprehensive coverage of the NHI in the Taiwanese population, the large case number, and the extensive adjustment for confounding factors in this study enhance the credibility of our findings. However, our study is also limited by the lack of laboratory data and the absence of AKI staging in the insurance claim database. The approach in this study, using in-patient AKI codes instead of using outpatient and in-patient codes in combination, might increase specificity for the recognition of AKI episodes at the expense of compromised sensitivity. Using administrative data to identify AKI episodes suffers from several drawbacks, including the lack of information on severity and etiology, and the use of non-consensus criteria for defining and staging AKI [30]. These issues are inherent to administrative data itself, contribute to an under-estimation of true AKI incidence, and are not amenable to correction currently. However, using administrative data to study AKI also has its advantage, including a higher specificity, the potential of amassing adequate case number to satisfy statistical requirement, and an efficient utilization of electronic medical records, which should not be overlooked according to the most recent Acute Dialysis Quality Initiative (ADQI) consensus statement [30]. Nonetheless, this under-estimation should affect users and non-users equally and thus might not significantly affect our findings. In addition, the AKI episodes we identified can include recurrent cases apart from incident ones, and the relationship between DPP4i use and the risk of developing AKI would be more complex if both types of episodes were included. Finally, results from the analyses focusing on the influence of different DPP4i doses on the risk of subsequent AKI can be substantially confounded by the required dosage adjustment in patients with declined renal function; thus, the interpretation of the dose-responsiveness relationship should be done with caution. Further validation of our results in a well-designed prospective cohort study is required, as well as a mechanistic study to elucidate the reasons for the beneficial effects of DPP4i on the risk of AKI.

## MATERIALS AND METHODS

### Ethical approval

The current study has been approved by the ethic committee of National Taiwan University Hospital (NO.201503028W). Consent from participants was waived due to the fact that data in the cohort have all been anonymized.

### Sources of patients and the database upon which clinical data were collected

This study was based on the NHI program, which has provided health care to the Taiwanese population since 1995. This program covers over 99% of the population, and the Bureau of NHI provides administrative data, constituting the National Health Insurance Research Database, which contains reimbursement information for all admissions, outpatient visits, and prescription records of all covered citizens. To protect patient privacy, the Bureau of NHI anonymizes the patient records and further scrambles the information for researchers.

### Study population

The study participant selection process is illustrated in Figure 1, and the medical and pharmacy records were obtained from all the diabetic Taiwanese citizens covered by Taiwan National Health Insurance” between 2007 and 2013. The definition of DM in our study cohort was based on the following criteria: having visited at least two separate outpatient clinics or having at least one admission with a diagnosis of DM according to the International Classification of Diseases (9th Revision, Clinical Modification [ICD-9-CM] code, 250.x); patients who met these criteria were enrolled in the study. The date of DM diagnosis described above was defined as the index date of entry into our study.

Since diabetic Taiwanese could not obtain DPP4is until 2008, when sitagliptin was first approved for use in Taiwan, we excluded participants with DM diagnosed before 2008 to identify incident DM patients, in order to prevent overestimating the risk of incident AKI and to ensure optimal patient selection. A falsely higher incidence of AKI might occur if we enrolled patients with DM diagnosed before 2008 due to their longer duration of having DM. In addition, patients diagnosed with DM after June 30, 2013, were also excluded to ensure a follow-up period of at least six months. We also excluded DM patients who already had ESRD before their designated index date. Due to an insufficient follow-up duration for linagliptin, which was approved in late 2012 in Taiwan, we did not include patients with DM who received linagliptin.

### Outcome definitions

The primary and secondary outcomes in this study were hospitalisations for AKI and dialysis-requiring AKI, respectively. To determine the primary outcome, we retrieved records from diabetic subjects diagnosed with AKI (ICD-9 CM code 580.x, 581.x, and 584.x) at admission after the date of DPP4i initiation [31]. Dialysis-requiring AKI was defined as the diagnosis of AKI at admission with procedure codes for dialysis at the same time. All patients were followed up from the index date until the date of outcome, death, or until December 31, 2013. Death was defined as the withdrawal of a subject from the NHI program, as validated in other studies [32].

### The assessment of DPP4i exposure

We reviewed the prescription records of the enrolled participants from the index date (DM diagnosis) until the date of event occurrence or the end of follow-up. The participants who did not receive DPP4i during the observation period were categorised as non-DPP4i users, while those who received any type of DPP4i for at least 90 consecutive days within a 365-day period after the first prescription date were categorised as DPP4i users. To clarify whether the influence of DPP4i on AKI was a class-effect or differed between each DPP4i member, we excluded patients receiving more than two types of DPP4i, including drug switching, during the observation period. The cumulative dosage of DPP4i was calculated as the total cumulative defined daily doses (DDDs). DDD is an assumed average daily maintenance dose of drug, as defined by the World Health Organization (WHO).

### Definitions of comorbidities, diabetic severity index, and other factors potentially influencing the risk of AKI

The comorbidity profiles of the study population were ascertained before the date of DM diagnosis, and included hypertension, hyperlipidaemia, liver disease, atrial fibrillation, chronic obstructive pulmonary disease, acute coronary syndrome, cerebrovascular disease, cancer, parkinsonism, CKD, advanced CKD (if CKD patients received erythropoietin concurrently [33]), past experiences of AKI, peripheral vascular disease, and benign prostatic hyperplasia, using codes listed in Supplementary Table 4. We also calculated the Charlson Comorbidity Index. The definition of comorbidity was verified if the participant was diagnosed during at least two outpatient clinics or at least once during any admission before the index date. Since medical interventions with potential renal influences may modify the risk of AKI, we also collected these patients’ histories of undergoing specific procedures, including computed tomography with contrast, cardiac catheterization, angiography of any sites, cystoscopy, and trans-urethral resection of the prostate, before the index date. Medications with influences on the risk of developing AKI were extracted based on the prescription records between the index date and the date of event occurrence or the end of follow-up, similar to the approach for assessing DPP4i exposure. Medication possession ratio (MPR) was calculated by assessing the number of days with medication available during the pre-specified period.

We also calculated an adapted Diabetes Complications Severity Index (aDCSI) and an aDCSI complication count for each participant enrolled in this study, in order to account for the severity of DM in the subsequent analyses, based on previously validated criteria in the literature [34, 35].

### Statistical analysis

A propensity score–matching approach was used to reduce the selection bias in this study. In addition, we also matched the entry date of the DPP4i users and non-users group, in order to decrease the possibility of immortal time bias between the two groups. The propensity score used in the reference group construction calculated the probability through a logistic regression model with covariates consisting of age, gender, residence, date of the first diagnosis of DM, renal risk-modifying interventions, comorbidities, and medications. We then used the propensity scores to match patients in the DPP4i user group with those in the non-user group at a 1:1 ratio. The randomly selected patients in the user group were matched to a non-user with the closest propensity score within the width of 0.01.

We first examined the baseline characteristics, including age, gender, residence, renal risk-modifying interventions, comorbidities, and medications among DPP4i users and matched non-users. Their demographic profiles and clinical characteristics were compared by chi-square and the Student's *t*-tests, respectively. We then calculated the incidence rate by dividing the numbers of AKI episodes by the total follow-up person-years (events per 1,000 person-years) within each group. We used the Kaplan-Meier method for AKI-free survival analysis, with a log-rank test for comparison between the DPP4i user and non-user groups. Univariate and multivariate regression analyses were subsequently utilised to estimate the hazard ratios (HRs) and 95% confidence intervals (CIs) based on Cox proportional hazards model in order to assess the risk of AKI due to DPP4i use. The non-user group served as the reference for comparison with each type of DPP4i in subgroup analyses. All models were adjusted for age, sex, residence, interventions, comorbidities, and medications used during the follow-up period. To further investigate the dose-response effects of DPP4i, users were divided into different strata of cumulative DDD. Sitagliptin users were divided into tertiles of cumulative DDD, while vildagliptin and saxagliptin users were divided into halves according to their median cumulative DDDs. The lowest dosage subgroup served as the reference for comparison. Finally, since a competing risk of death should be considered when the study outcome were AKI, we also incorporated mortality as a competing risk in our sensitivity analysis.

All statistical tests were two-sided and *P*-values < 0.05 were considered statistically significant. Statistical analyses were performed using SAS version 9.4 (SAS Institute, Cary, NC).

## CONCLUSIONS

Using data from a nationwide population-based cohort, we found that the use of DPP4i (sitagliptin, vildagliptin, and saxagliptin) was associated with a reduced risk of incident AKI after treatment initiation, regardless of age, presence of CKD, or history of AKI. These findings shed further light on the potential renal effect of DPP4i.

## SUPPLEMENTARY FIGURE AND TABLES


