# Supplementary material for: Dipeptidyl peptidase 4 inhibitor use is associated with a lower risk of incident acute kidney injury in patients with diabetes
Source: Oncotarget. 2017 May 23;8(32):53028–40. doi: 10.18632/oncotarget.18081 (PMC5581090; doi:10.18632/oncotarget.18081)
Supplement: Supplementary file 1 [file oncotarget-08-53028-s001.pdf]

Dipeptidyl peptidase 4 inhibitor use is associated with a lower risk of incident acute kidney injury in patients with diabetes

Supplementary Materials

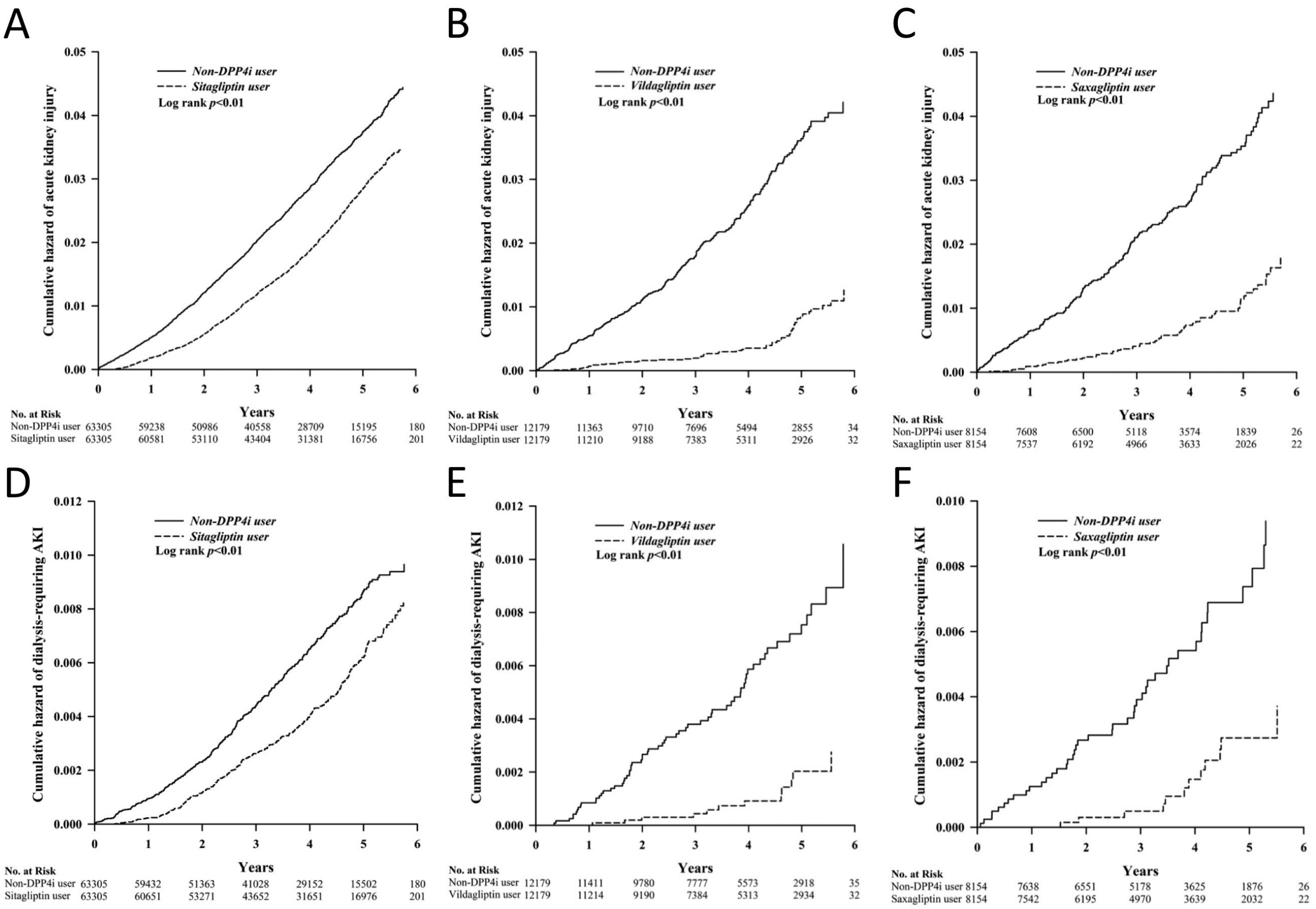

Supplementary Figure 1: Kaplan-Meier cumulative hazard curve for incident AKI among non-DPP4i users and sitagliptin (**A**), vildagliptin (**B**), and saxagliptin (**C**) users, and for incident dialysis-requiring AKI among non-DPP4i users and sitagliptin (**D**), vildagliptin (**E**), and saxagliptin (**F**) users. AKI, acute kidney injury; DPP4i, dipeptidyl peptidase 4 inhibitor.

Supplementary Table 1: Incidence and risk of acute kidney injury among diabetic participants, based on the presence of CKD or not

	Without CKD							With CKD						
				Crude analysis†		Fully adjusted Model*							Fully adjusted Model*	
Variables	Number of event	Duration (person-years)	Incidence density	HR	95% CI	HR	95% CI	Number of event	Duration (person-years)	Incidence density	HR	95% CI	HR	95% CI
Incidence of acute kidney injury														
Non-DPP4i users	439	25174.3	17.4	1.00	-	1.00	-	1679	274625.8	6.1	1.00	-	1.00	-
DPP4i users	286	25745.2	11.1	0.64	0.55 ~ 0.75[a]	0.56	0.48 ~ 0.65[a]	1018	283883.5	3.6	0.58	0.54 ~ 0.63[a]	0.57	0.53 ~ 0.62[a]
Non-DPP4i users	331	19280.5	17.2	1.00	-	1.00	-	1292	208232.7	6.2	1.00	-	1.00	-
Sitagliptin users	248	19618.7	12.6	0.73	0.62 ~ 0.87[a]	0.64	0.54 ~ 0.75[b]	943	218759.9	4.3	0.69	0.63 ~ 0.75[a]	0.67	0.61 ~ 0.73[a]
Non-DPP4i users	62	3528.7	17.6	1.00	-	1.00	-	229	39925.6	5.7	1.00	-	1.00	-
Vildagliptin users	16	3371.9	4.7	0.27	0.16 ~ 0.47[a]	0.22	0.12 ~ 0.40[a]	38	38988.4	1.0	0.17	0.12 ~ 0.24[a]	0.17	0.12 ~ 0.25[a]
Non-DPP4i users	46	2365.1	19.4	1.00	-	1.00	-	158	26467.5	6.0	1.00	-	1.00	-
Saxagliptin users	22	2457.8	9.0	0.46	0.28 ~ 0.76[b]	0.39	0.22 ~ 0.67[b]	37	26135.2	1.4	0.24	0.16 ~ 0.34[a]	0.24	0.17 ~ 0.35[a]
Incident dialysis-requiring acute kidney injury														
Non-DPP4i users	111	25745.2	4.3	1.00	-	1.00	-	358	283883.5	1.3	1.00	-	1.00	-
DPP4i users	63	25696.5	2.5	0.57	0.42 ~ 0.77[a]	0.50	0.36 ~ 0.69[a]	226	284661.9	0.8	0.61	0.51 ~ 0.72[a]	0.60	0.51 ~ 0.71[a]
Non-DPP4i users	86	19685.8	4.4	1.00	-	1.00	-	277	209678.0	1.3	1.00	-	1.00	-
Sitagliptin users	56	19851.1	2.8	0.64	0.46 ~ 0.90[b]	0.55	0.39 ~ 0.78[b]	209	219511.6	1.0	0.71	0.59 ~ 0.85[a]	0.70	0.59 ~ 0.84[a]
Non-DPP4i users	19	3625.4	5.2	1.00	-	1.00	-	44	40182.1	1.1	1.00	-	1.00	-
Vildagliptin users	5	3376.8	1.5	0.28	0.11 ~ 0.76[c]	0.21	0.07~0.65[b]	7	39000.1	0.2	0.17	0.07 ~ 0.37[a]	0.17	0.08 ~ 0.39[a]
Non-DPP4i users	6	2434.0	2.5	1.00	-	1.00	-	37	26629.7	1.4	1.00	-	1.00	-
Saxagliptin users	2	2468.6	0.8	0.34	0.07 ~ 1.67	0.01	0.00 ~ 1.61	10	26150.2	0.4	0.27	0.14 ~ 0.55[b]	0.29	0.14 ~ 0.59[b]

[a]$p < 0.0001$; [b]$p < 0.01$; [c]$p < 0.05$
† Calculated by stratified Cox proportional regression
* Adjusted for age, gender, all comorbidities, and medications
CI, confidence interval; CKD, chronic kidney disease; DPP4i, dipeptidyl peptidase 4 inhibitor; HR, hazard ratio.

Supplementary Table 2: Incidence and risk of acute kidney injury among diabetic participants, based on the presence of past AKI or not

	Without past history of AKI							With past history of AKI						
				Crude analysis†		Fully adjusted Model*					Crude analysis†		Fully adjusted Model*	
**Variables**	**Number of event**	**Duration (person-years)**	**Incidence density**	**HR**	**95% CI**	**HR**	**95% CI**	**Number of event**	**Duration (person-years)**	**Incidence density**	**HR**	**95% CI**	**HR**	**95% CI**
**Incidence of acute kidney injury**														
**Non-DPP4i users**	**98**	**2587.2**	**37.9**	**1.00**	**-**	**1.00**	**-**	**2020**	**297212.9**	**6.8**	**1.00**	**-**	**1.00**	**-**
DPP4i users	52	2362.6	22.0	0.57	0.41 ~ 0.80[b]	0.50	0.35 ~ 0.72[b]	1252	306969.4	4.1	0.60	0.55 ~ 0.64[a]	0.57	0.53 ~ 0.62[a]
Non-DPP4i users	73	1991.2	36.7	1.00	-	1.00	-	1550	225522.0	6.9	1.00	-	1.00	-
Sitagliptin users	46	1843.6	25.0	0.67	0.47 ~ 0.97[c]	0.53	0.36 ~ 0.78[b]	1145	236535.1	4.8	0.70	0.65 ~ 0.75[a]	0.67	0.62 ~ 0.72[a]
Non-DPP4i users	12	347.6	34.5	1.00	-	1.00	-	279	43106.7	6.5	1.00	-	1.00	-
Vildagliptin users	3	259.2	11.6	0.31	0.09 ~ 1.11	-	-	51	41101.1	1.2	0.19	0.14 ~ 0.25[a]	0.19	0.14 ~ 0.25[a]
Non-DPP4i users	13	248.4	52.3	1.00	-	1.00	-	191	28584.2	6.7	1.00	-	1.00	-
Saxagliptin users	3	259.8	11.5	0.21	0.06 ~ 0.74[c]	-	-	56	28333.2	2.0	0.29	0.22 ~ 0.40[a]	0.28	0.21 ~ 0.38[a]
Incident dialysis-requiring acute kidney injury														
Non-DPP4i users	26	2711.8	9.6	1.00	-	1.00	-	443	299523.2	1.5	1.00	-	1.00	-
DPP4i users	13	2410.2	5.4	0.57	0.29 ~ 1.10	0.50	0.24 ~ 1.03	276	307948.2	0.9	0.60	0.52 ~ 0.70[a]	0.57	0.49 ~ 0.67[a]
Non-DPP4i users	19	2069.3	9.2	1.00	-	1.00	-	344	227294.4	1.5	1.00	-	1.00	-
Sitagliptin users	12	1887.2	6.4	0.70	0.34 ~ 1.45	0.53	0.23 ~ 1.23	253	237475.5	1.1	0.70	0.59 ~ 0.82[a]	0.66	0.56 ~ 0.78[a]
Non-DPP4i users	6	364.7	16.5	1.00	-	1.00	-	57	43442.9	1.3	1.00	-	1.00	-
Vildagliptin users	1	259.3	3.9	0.21	0.03 ~ 1.78	-	-	11	42117.5	0.3	0.20	0.11 ~ 0.38[a]	0.20	0.10 ~ 0.38[a]
Non-DPP4i users	1	277.8	3.6	1.00	-	1.00	-	42	28785.9	1.4	1.00	-	1.00	-
Saxagliptin users	0	263.6	0.0	-	-	-	-	12	28355.2	0.4	0.29	0.15 ~ 0.55[b]	0.31	0.16 ~ 0.59[b]

[a] $p < 0.0001$; [b] $p < 0.01$; [c] $p < 0.05$

† Calculated by stratified Cox proportional regression

* Adjusted for age, gender, all comorbidities, and medications

AKI, acute kidney injury; CI, confidence interval; DPP4i, dipeptidyl peptidase 4 inhibitor; HR, hazard ratio

Supplementary Table 3: Incidence and risk of acute kidney injury among diabetic participants, based on their age category

	Under 64 years							≥ 65 years						
				Crude analysis†		Fully adjusted Model*					Crude analysis†		Fully adjusted Model*	
**Variables**	**Number of event**	**Duration (person-years)**	**Incidence density**	**HR**	**95% CI**	**HR**	**95% CI**	**Number of event**	**Duration (person-years)**	**Incidence density**	**HR**	**95% CI**	**HR**	**95% CI**
Incidence of acute kidney injury														
Non-DPP4i users	1223	231437.5	5.3	1.00	-	1.00	-	895	68362.5	13.1	1.00	-	1.00	-
DPP4i users	675	239117.7	2.8	0.53	0.48 ~ 0.58[a]	0.53	0.48 ~ 0.58[a]	629	70214.2	9.0	0.68	0.62 ~ 0.75[a]	0.63	0.57 ~ 0.70[a]
Non-DPP4i users	912	174825.5	5.2	1.00	-	1.00	-	711	52687.7	13.5	1.00	-	1.00	-
Sitagliptin users	620	183517.5	3.4	0.64	0.58 ~ 0.71[a]	0.64	0.58 ~ 0.71[a]	571	54861.1	10.4	0.77	0.69 ~ 0.86[a]	0.71	0.63 ~ 0.79[a]
Non-DPP4i users	183	34396.7	5.3	1.00	-	1.00	-	108	9057.6	11.9	1.00	-	1.00	-
Vildagliptin users	28	33555.5	0.8	0.16	0.11 ~ 0.23[a]	0.15	0.10 ~ 0.23[a]	26	8804.8	3.0	0.25	0.16 ~ 0.38[a]	0.22	0.14 ~ 0.35[a]
Non-DPP4i users	128	22215.3	5.8	1.00	-	1.00	-	76	6617.3	11.5	1.00	-	1.00	-
Saxagliptin users	27	22044.7	1.2	0.21	0.14 ~ 0.32[a]	0.21	0.14 ~ 0.33[a]	32	6548.3	4.9	0.43	0.28 ~ 0.64[a]	0.36	0.23 ~ 0.56[a]
Incident dialysis-requiring acute kidney injury														
Non-DPP4i users	282	232959.3	1.2	1.00	-	1.00	-	187	69275.7	2.7	1.00	-	1.00	-
DPP4i users	184	239682.6	0.8	0.63	0.52~0.76[a]	0.63	0.52~0.76[a]	105	70675.8	1.5	0.55	0.43 ~ 0.70[a]	0.49	0.39 ~ 0.63[a]
Non-DPP4i users	213	175951.4	1.2	1.00	-	1.00	-	150	53412.4	2.8	1.00	-	1.00	-
Sitagliptin users	165	184065.5	0.9	0.73	0.60 ~ 0.89[b]	0.73	0.60 ~ 0.90[b]	100	55297.2	1.8	0.64	0.50 ~ 0.83[b]	0.57	0.44 ~ 0.74[a]
Non-DPP4i users	42	34634.9	1.2	1.00	-	1.00	-	21	9172.6	2.3	1.00	-	1.00	-
Vildagliptin users	10	33562.5	0.3	0.25	0.12 ~ 0.49[a]	0.27	0.13~0.64[b]	2	8814.3	0.2	0.10	0.02 ~ 0.43[b]	0.05	0.01 ~ 0.27[b]
Non-DPP4i users	27	22373.0	1.2	1.00	-	1.00	-	16	6690.7	2.4	1.00	-	1.00	-
Saxagliptin users	9	22054.6	0.4	0.34	0.16 ~ 0.71[b]	0.01	0.17 ~ 0.79[c]	3	6564.3	0.5	0.19	0.06 ~ 0.67[b]	0.16	0.04 ~ 0.58[b]

[a] $p < 0.0001$; [b] $p < 0.01$; [c] $p < 0.05$
† Calculated by stratified Cox proportional regression
* Adjusted for age, gender, all comorbidities, and medications
CI, confidence interval; DPP4i, dipeptidyl peptidase 4 inhibitor; HR, hazard ratio

Supplementary Table 4: ICD-9-CM codes of comorbidities extracted

Comorbidities	ICD-9-CM codes
Hypertension	401.x ~ 405.x
Hyperlipidemia	272.x
Chronic liver disease	570.x ~ 573.x
Atrial fibrillation	427.x
Chronic obstructive pulmonary disease	491.x
Acute coronary syndrome	410.x, 411.x, 414.x
Cerebrovascular disease	430.x ~ 438.x
Malignancy	140.x ~ 208.x
Parkinsonism	332.x
Chronic kidney disease	016.0x, 042.x, 095.4, 189.x, 223.x, 236.9x, 250.4x, 271.4, 274.1x, 403.x, 404.x, 440.1, 442.1, 446.21, 447.3x, 572.4, 580.x ~ 589.x, 590.x, 591.x, 593.x, 642.1x, 646.2x, 753.x, 984.x
Acute kidney injury	580.x, 581.x, 584.x
Peripheral vascular disease	441.x, 443.9x, 785.x, V434, ICD-9-CM Procedure Code 38.49
Benign prostatic hyperplasia	600.0x
